# Spatiotemporal Shift of T4-Like Phage Community Structure in the Three Largest Estuaries of China

**DOI:** 10.1128/spectrum.05203-22

**Published:** 2023-03-06

**Authors:** Lanlan Cai, Bu Xu, Huifang Li, Yongle Xu, Wei Wei, Rui Zhang

**Affiliations:** a State Key Laboratory of Marine Environmental Science, College of Ocean and Earth Sciences, Xiamen University, Xiamen, China; b Department of Ocean Science, The Hong Kong University of Science and Technology, Hong Kong, China; c School of Environment, Harbin Institute of Technology, Harbin, China; d Department of Ocean Science and Engineering, Southern University of Science and Technology, Shenzhen, China; e Key Laboratory of Coastal Salt Marsh Ecosystems and Resources, Ministry of Natural Resources, Jiangsu Ocean University, Lianyungang, China; f Institute of Marine Science and Technology, Shandong University, Shandong, China; g School of Environmental Ecology and Biological Engineering, Wuhan Institute of Technology, Wuhan, China; h Institute for Advanced Study, Shenzhen University, Shenzhen, China; Nanjing Institute of Geography and Limnology Chinese Academy of Sciences

**Keywords:** viral community, Chinese estuary, T4-like bacteriophage, g23, seasonality

## Abstract

Estuaries are one of the most highly productive and economically important ecosystems at the continent-ocean interface. Estuary productivity is largely determined by the microbial community structure and activity. Viruses are major agents of microbial mortality and are key drivers of global geochemical cycles. However, the taxonomic diversity of viral communities and their spatial-temporal distribution in estuarine ecosystems have been poorly studied. In this study, we investigated the T4-like viral community composition at three major Chinese estuaries in winter and in summer. Diverse T4-like viruses, which were divided into three main clusters (Clusters I to III), were revealed. The Marine Group of Cluster III, with seven identified subgroups, was the most dominant (averaging 76.5% of the total sequences) in the Chinese estuarine ecosystems. Significant variations of T4-like viral community composition were observed among estuaries and seasons, with higher diversity occurring in winter. Among various environmental variables, temperature was a main driver of the viral communities. This study demonstrates viral assemblage diversification and seasonality in Chinese estuarine ecosystems.

**IMPORTANCE** Viruses are ubiquitous but largely uncharacterized members of aquatic environments that cause significant mortality in microbial communities. Recent large-scale oceanic projects have greatly advanced our understanding of viral ecology in marine environments, but those studies mostly focused on oceanic regions. There have yet to be spatiotemporal studies of viral communities in estuarine ecosystems, which are unique habitats that play a significant role in global ecology and biogeochemistry. This work is the first comprehensive study that provides a detailed picture of the spatial and seasonal variation of viral communities (specifically, T4-like viral communities) in three major estuarine ecosystems in China. These findings provide much-needed knowledge regarding estuarine viral ecosystems, which currently lags in oceanic ecosystem research.

## INTRODUCTION

The importance of estuaries in the flux of matter from terrestrial to marine environments and their proximity to major urban areas have made estuarine ecosystems a key focus of the natural and social sciences (1–3). Estuaries often exhibit strong spatiotemporal gradients in salinity, turbidity, and nutrients. This leads to a metabolically versatile group of microorganisms that are adapted to the environments being the main drivers of biogeochemical cycling (1, 4). Microbial community activity is essentially mediated via top-down (e.g., predation and viral lysis) and bottom-up (e.g., resource availability) controls (5). Viruses, one of the major top-down control factors for microbes, are numerically abundant and genetically diverse components of aquatic ecosystems (6). They are important agents of microbial mortality and are estimated to lyse 20% to 40% of marine microbes daily, releasing cellular carbon and nutrients that increase the resource availability for surviving cells and strongly impact the entire food web (6, 7). Beyond mortality, viruses can exert selective pressure on the evolution of their hosts and alter biogeochemical processes by metabolically reprogramming host metabolisms, such as photosynthesis, carbon metabolism, and the nitrogen and sulfur cycling pathways (7, 8). Thus, viral-mediated host mortality and nutrient availability are critical to the microbial composition and biogeochemical cycles in estuarine ecosystems, and the magnitude of viral impact varies temporally and spatially. Despite the ecological and evolutionary importance of viruses, the spatiotemporal distribution and environmental constraints of viral community composition in estuarine ecosystems are poorly delineated and are currently lagging behind oceanic research.

T4-like viruses (which are phages that share genetic homologies and morphological similarities with the model coliphage T4) are one of the most widespread and diverse viral groups in aquatic, soil, and sediment environments (9–12). They infect a broad range of bacteria, including various genera of heterotrophic Proteobacteria (e.g., Escherichia coli, *Shigella*, *Vibrio*, Salmonella, SAR11) and autotrophic cyanobacteria, which are fundamental components of microbial communities (11, 13, 14). Phages within the T4-like subfamily typically share a core genome of 31 to 33 genes, among which *g23* encodes the major capsid protein (10, 11, 15). To date, the diversity of the T4-like viruses has been investigated in various environments (e.g., seawater, lake, paddy water, rice field, wetland sediments); their prevalence across environments indicates that they play an important role in the diversity, community structure, evolution, and function of ecosystems (16–22). For example, infection by T4-like cyanophages inhibits CO_2_ fixation in cyanobacteria (23, 24); the T4-like viral lysis of the ubiquitous cyanobacteria and SAR11 releases cell contents to the organic carbon pool and accelerates carbon cycling in aquatic ecosystems (25). In addition, T4-like cyanophages drive photosystem evolution through their antagonistic interactions and/or introduction of new genetic information to bacteria (6, 23, 26–30).

**FIG 1:**
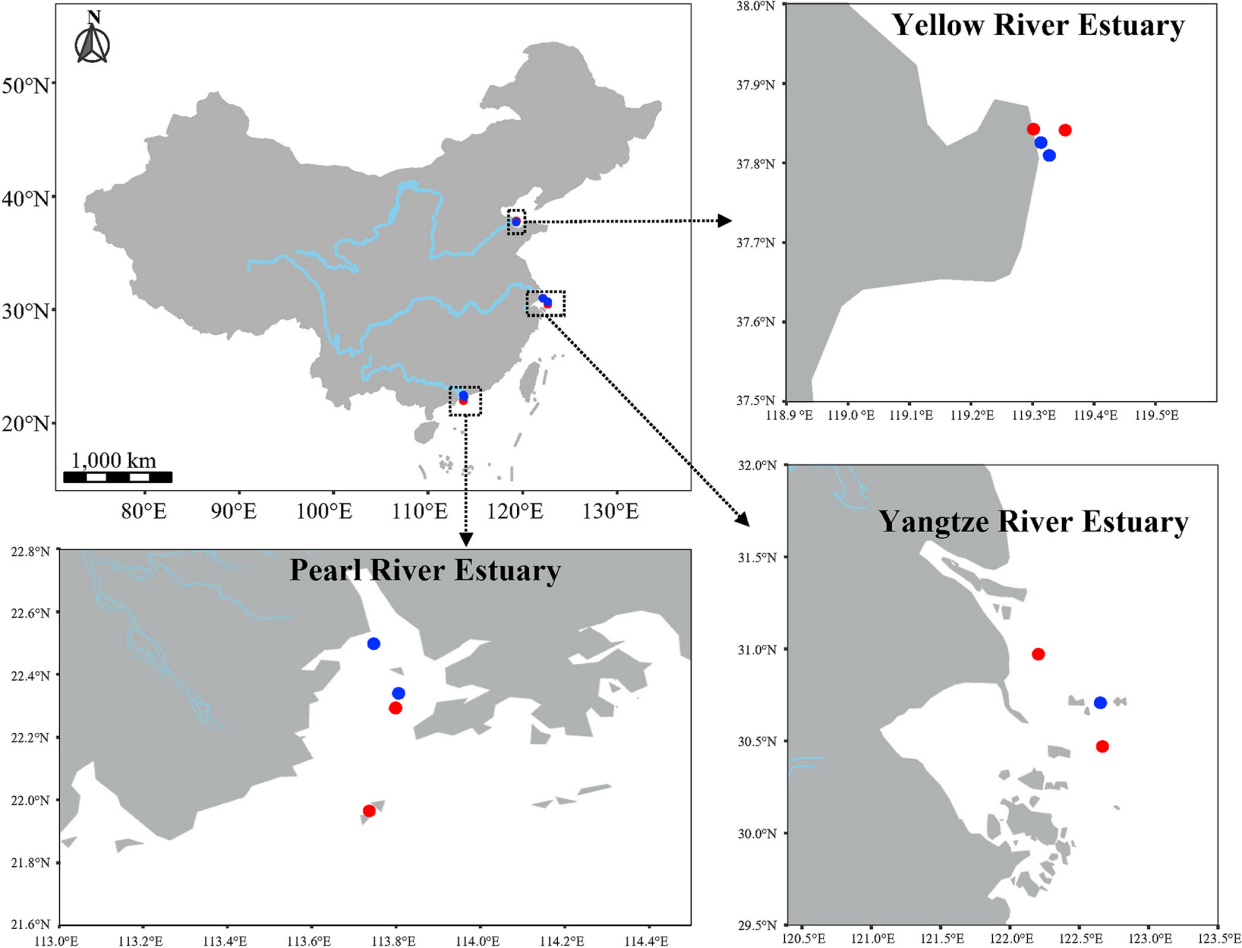
Locations of the sampling stations in three major Chinese estuaries. The sampling sites (points) are colored based on the sampling season (blue for winter and red for summer).

The estuaries along the coast of China cover a region between 5 to 55°N latitude, ranging from tropical to cold temperate climates. The diversity of viral communities in Chinese estuaries has been examined through a few studies at individual estuaries at a single time. For example, viral diversity has been investigated in the Jiulong River Estuary, revealing diverse and novel estuarine viral groups that adapted to local environments (18, 31). However, numerous studies have suggested that viral communities temporally and spatially vary (17, 32, 33); and there have not been any spatiotemporal studies of viral communities in estuarine ecosystems. Therefore, in this study, we conducted a systematic investigation of the Chinese estuarine T4-like viral community at the three largest Chinese estuaries, which are located up to 2,000 km apart, in two different seasons (summer and winter) (Fig. 1). The objectives of this study were to: (i) elucidate T4-like viral community structures and their seasonal dynamics in Chinese estuarine ecosystems and (ii) search for possible environmental factors that are involved in the structuring of the viral community structures. To our knowledge, this is the first integrated investigation of the seasonality of viral diversity across estuaries that provides a spatiotemporal insight into T4-like bacteriophage diversity and their biogeographic distribution in Chinese estuarine ecosystems.

## RESULTS

### Diversity of the estuarine T4-like viral communities.

Field studies focusing on T4-like viral communities were conducted at three major estuaries along the coast of China (Fig. 1), including the subtropical Pearl River Estuary (PRE), subtropical Yangtze River Estuary (YZRE), and temperate Yellow River Estuary (YRE). After quality filtering, denoising, and chimera removal, a total of 229,364 high-quality *g23* sequences, varying from 6,696 to 25,826 per sample, were obtained from Chinese estuaries. Approximately 90.8% of sequences (208, 264) were assigned to 425 OTUs at a 97% similarity level. The richness oscillated between 31 and 159 OTUs per sample, with the lowest being at the mesohaline zone of YRE in summer and the highest being at the mesohaline zone of YRE in winter (Fig. S1A). Rarefaction curves related to the detected OTUs showed that most samples reached saturation (Fig. S1A). A rarefaction curve for the pooled data from all samples was also generated to produce a global view of the diversity by considering the estuary as a single environment (Fig. S1B). The curve showed a clear sign of saturation, with a maximum richness (425 OTUs) being estimated at around 50,000 sequences, thereby providing an estimate of the global number of T4-like virus OTUs in the Chinese estuarine ecosystems. Samples were then subsampled to 6,146 sequences per sample (the minimum number of sequences of all the of samples) for the downstream analyses. The final OTU table (425 OTUs and 67,606 sequences) was used to analyze the taxonomic affiliations of estuarine T4-like viruses and their spatiotemporal dynamics.

**FIG 2:**
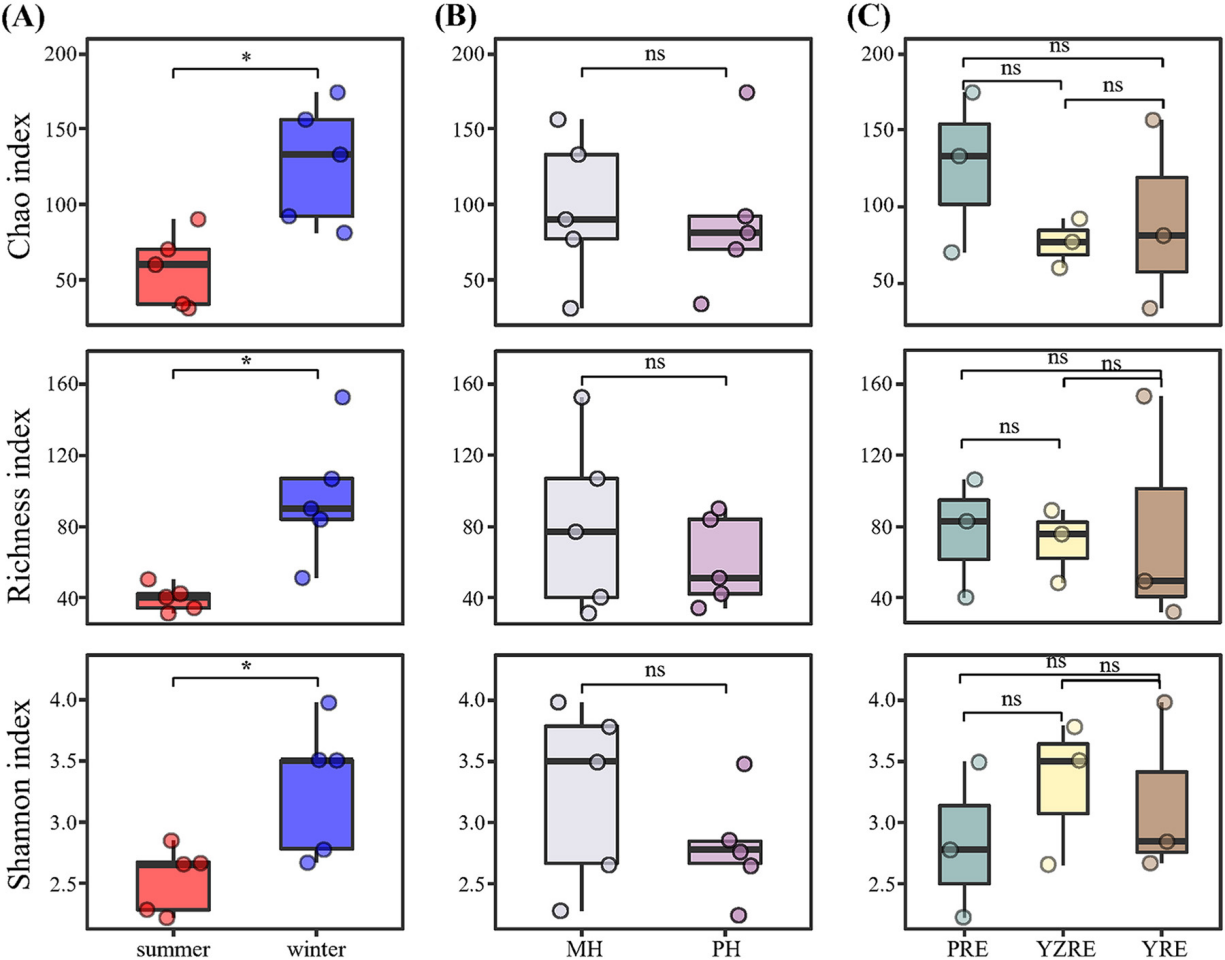
The comparison of *α*-diversity across different seasons (A), regions with different salinity levels (B), and estuaries (C). The statistical significance of the differences between two groups was tested using a paired *t* test. *, *P* < 0.05; ns (no significance), *P* > 0.05. MH, mesohaline zone; PH, polyhaline zone; PRE, Pearl River Estuary; YZRE, Yangtze River Estuary; YRE, Yellow River Estuary.

The T4-like viral complexity in different samples was estimated on the basis of *α*-diversity indices, including the Chao, Richness, and Shannon indices. The Chao, Richness, and Shannon indices in winter were all significantly higher than those in summer (*P* < 0.05, *t* test) (Fig. 2A). Correspondingly, the Richness and Shannon indices were negatively correlated with prokaryotic abundance and temperature (*P* < 0.05) (Fig. S2). No significant differences in α-diversity were found across estuaries and regions with different salinity levels (*P* > 0.05) (Fig. 2B and C).

### Spatiotemporal pattern of the estuarine T4-like viral communities.

**FIG 3:**
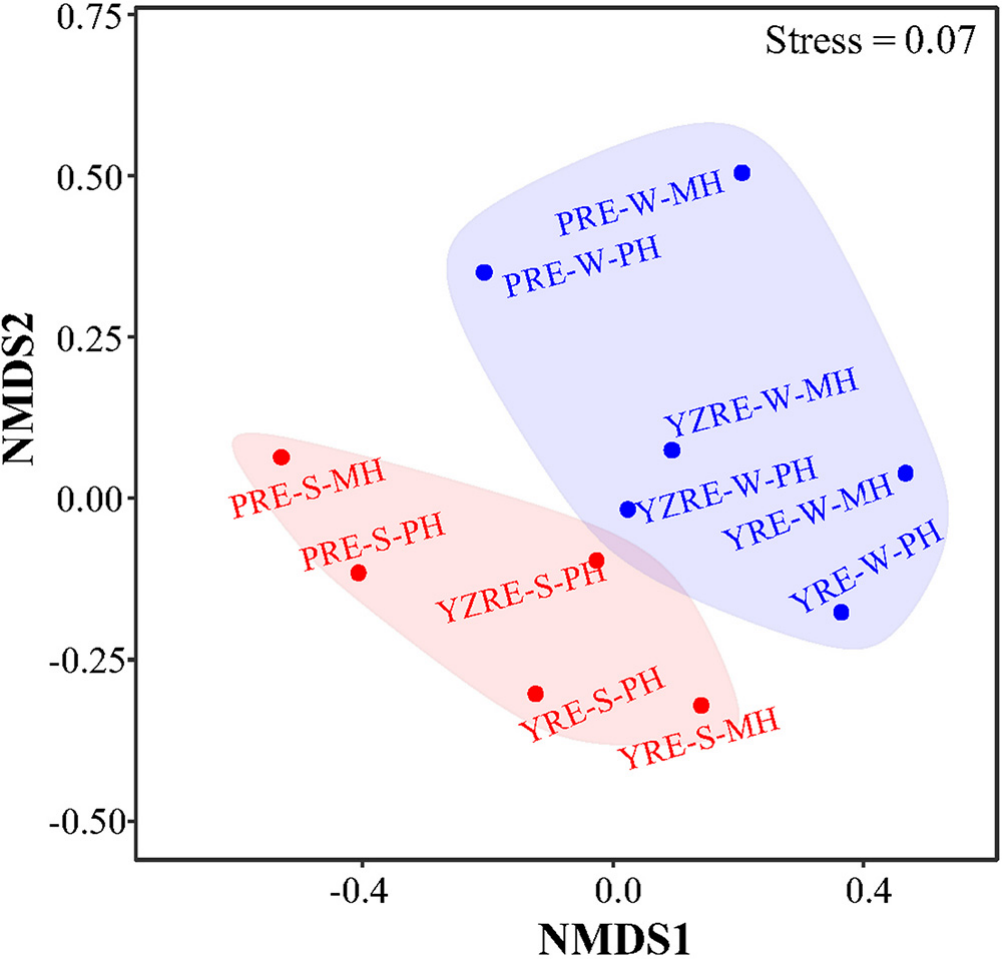
Nonmetric multidimensional scaling (NMDS) plot showing the variation in the T4-like viral community composition between seasons. The pairwise community distances were determined using Bray-Curtis dissimilarities at the OTU level. The stress level is indicated. The samples (points) are colored based on the sampling season (blue for winter and red for summer). Significant differences between seasons were verified via a PERMANOVA (*P* ≤ 0.001).

The differences in T4-like virus community composition between samples were explored using Bray-Curtis dissimilarities. The observed dissimilarities across Chinese estuarine samples ranged from 0.671 to 0.997, which indicated high compositional dissimilarity between the samples and the dynamics in the viral community. A nonmetric multidimensional scaling (NMDS) plot was used to visualize the similarity among different samples. The analysis showed a tendency for seasonal separation, with samples clustering into the summer and winter groups, respectively (Fig. 3). Moreover, we observed a striking separation of communities for the three studied estuaries, with the level of similarity among the three estuaries mirroring their geographic distance (i.e., a clear trend from PRE to YZRE and YRE) (Fig. S3). The seasonal and estuarine separations of T4-like virus composition were further supported by a PERMANOVA (*P ≤ *0.001 and *P* < 0.05). However, the sampling regions with different salinity levels did not show a distinct clustering pattern (*P* > 0.05). To further explore the mechanisms shaping the viral biogeographic patterns, the influence of environmental variables (e.g., temperature, salinity, dissolved oxygen, microbial abundances, viral production) in forming viral community structures was analyzed. A Mantel test showed that the temperature was significantly correlated with the viral community composition (*P* < 0.05).

### Taxonomic composition of the estuarine T4-like viral communities.

**FIG 4:**
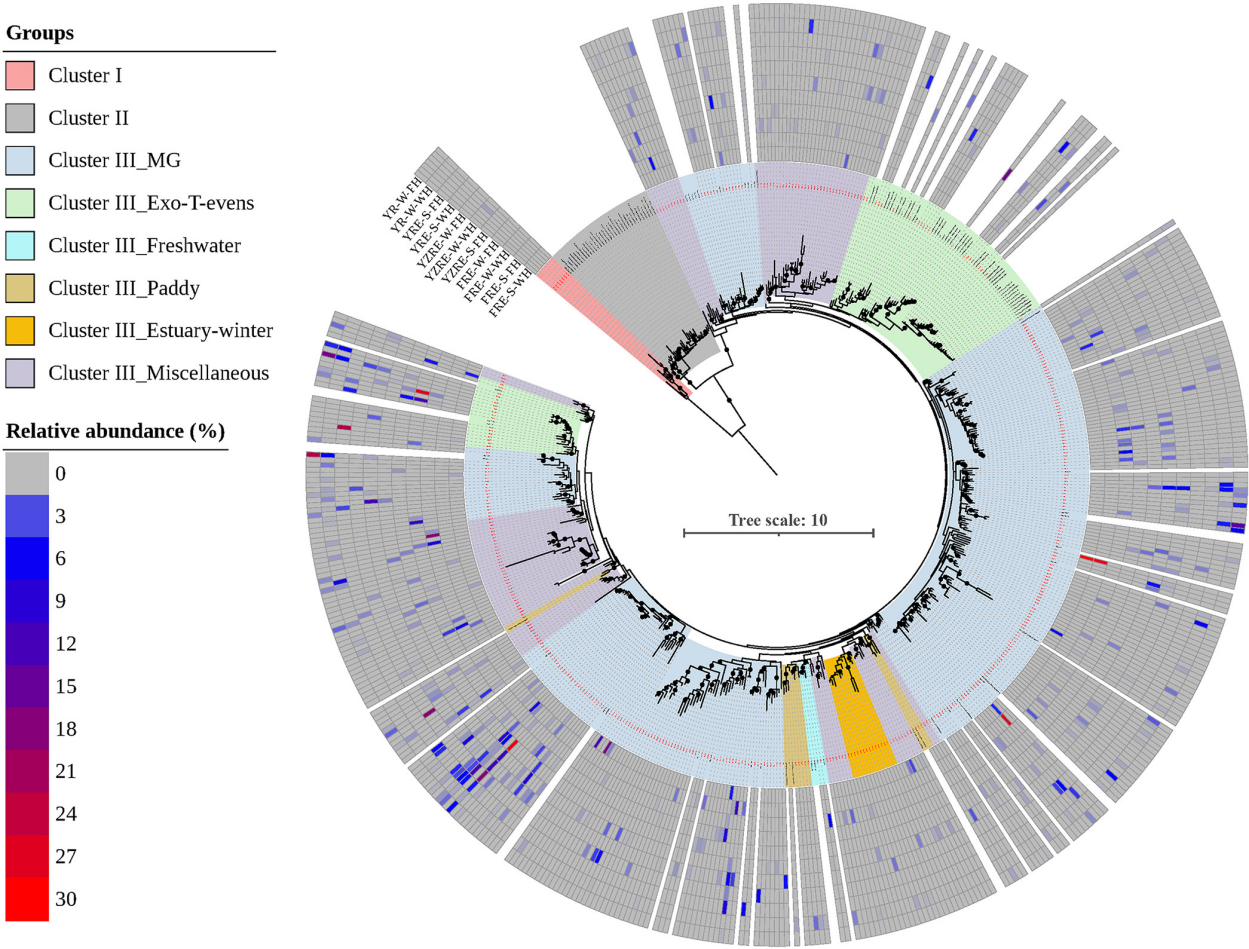
Maximum likelihood phylogenetic tree based on *g23* amino acid sequences. Different colored ranges indicate *g23* sequences from different groups or origins. Black dots indicate internal nodes with a bootstrap support of >50% (1,000 replicates). The outer colored rings indicate the relative abundance of sequences of each OTU in each sample. The OTUs that were obtained in this study are shown in red.

A BLAST analysis of amino acid sequences showed that the majority of the *g23* OTUs (400 out of 425) in the three largest Chinese estuaries showed the closest identities with those of the uncultured T4-like viruses. This indicated a large body of uncharacterized T4-like viruses in nature. All of the *g23* OTUs obtained in Chinese estuaries, the representative *g23* genes from different environments (e.g., marine, estuary, lake, paddy) (9, 12, 17, 21, 22), and the *g23* genes of isolated T4-like phages in the NCBI NR database were used to build a phylogenetic tree. Overall, the phylogenetic profiles of the *g23* genes formed three distinct clusters, which are referred to as Clusters I to III (Fig. 4). Cluster I solely consisted of seven OTUs from this study, indicating a novel clade that is unique to Chinese estuaries. Cluster II was from phages belonging to the previously defined T-even, Pseudo T-even, and Schizo T-even groups, the hosts of which primarily inhabit animal guts (11, 34, 35). No OTUs from Chinese estuaries fell into Cluster II. Most of the OTUs (418 out of 425) that were obtained in this study fell into Cluster III, which included *g23* sequences that were retrieved from different environments and isolated cyanophages.

**FIG 5:**
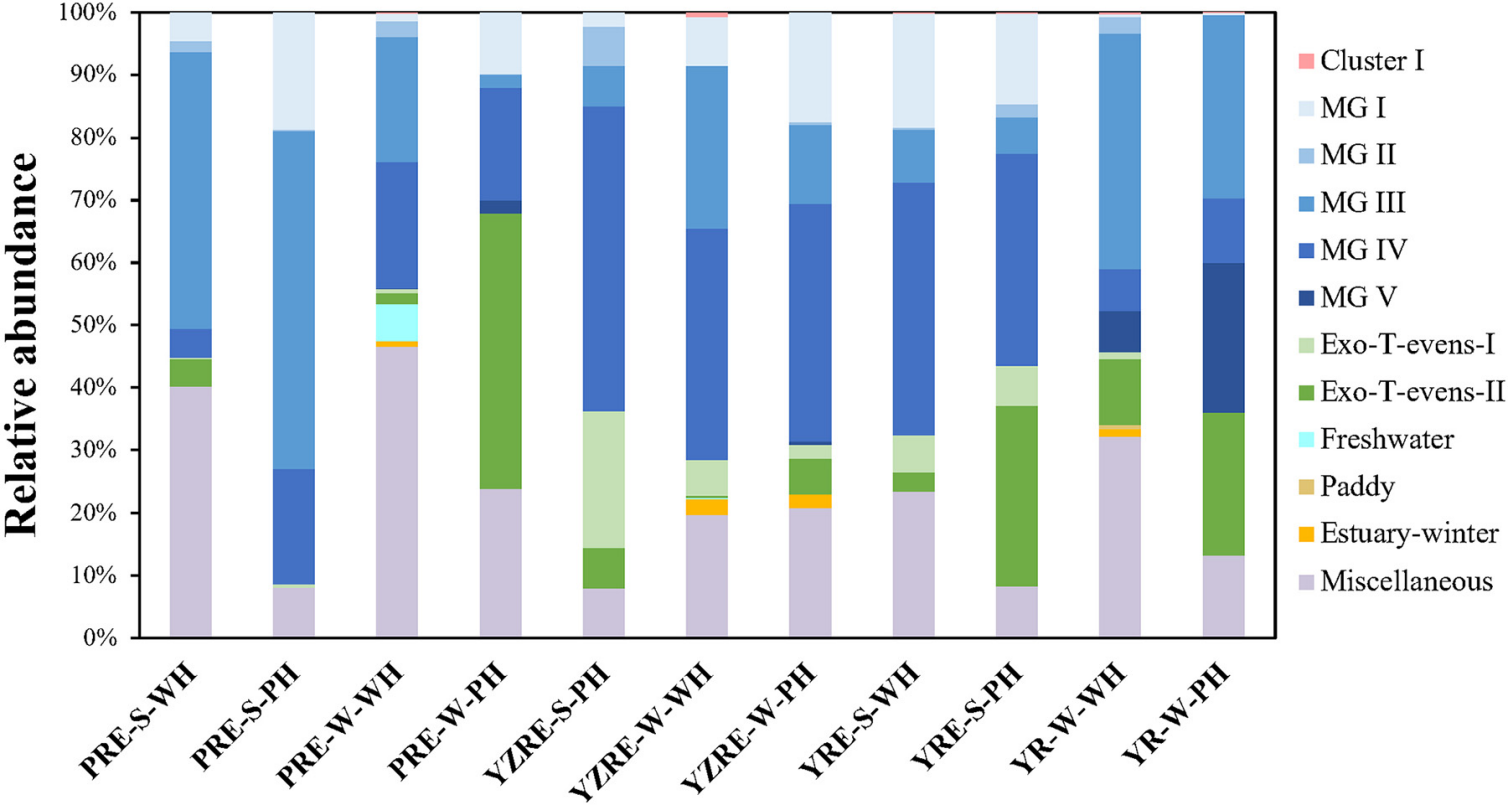
The distribution of *g23* sequences of OTUs in different subgroups in each sample. Different colors indicate *g23* sequences from different groups or origins. Red indicates sequences that are unique to Chinese estuaries. Blue indicates the Marine Group I–V, which is a group of sequences from the marine environment. Green indicates Exo-T-evens, a group consisting of T4-like viruses that infect cyanobacteria. Sequences that originate from freshwater are in light cyan, and those from paddies are in brown. Purple indicates a group that contained sequences from various habitats.

In the Chinese estuarine ecosystems, the OTUs within Cluster I only existed in a few samples and accounted for 0.1% to 0.7% of the sequences per sample. Therefore, Cluster I represented a group of T4-like viruses that is unique and rare in the Chinese estuarine environments (Fig. 4 and 5). According to the clustering with the sequences from different environments, Cluster III could be divided into different groups, including the Marine Group (MG), Freshwater Group, Paddy Group, and Miscellaneous Group (a group containing sequences from various habitats) (Fig. 4). The most abundant T4-like viruses of the Chinese estuarine ecosystems belonged to MG, which could be further subdivided into five MG subgroups (I to V) and two subgroups of Exo-T-even viruses that were predicted to infect cyanobacteria (Exo-T-evens–I and Exo-T-evens–II). Previous studies showed that MG I and MG IV had wide geographical distributions, whereas MG II, MG III, and MGV were abundant in low-salinity eutrophic environments (9, 17). Sequences of the Exo-T-evens–I group were more closely related to the *g23* genes of isolated cyanophages. The Exo-T-evens–II group contained environmental-only sequences that were mainly derived from marine environments (9, 17, 20). MG sequences accounted for 59.8% to 92.0% (average, 76.5%) of the sequences in different samples, with the relative abundances of subgroups varying among samples (Fig. 5).

The most abundant T4-like viruses in the subtropical estuary YZRE, regardless of the different seasons and sampling regions with different salinity levels, were all affiliated with MG IV and accounted for 37.0% to 48.7% of the sequences per sample. For the temperate estuary YRE, MG IV was the most abundant subgroup in summer (40.4% and 33.9% of the sequences in mesohaline and polyhaline zones, respectively), whereas MG III was most prevalent in winter (37.6% and 29.3% of sequences in the mesohaline and polyhaline zones, respectively). In contrast, for the subtropical estuary PRE, the sequences affiliated with MG III showed the highest relative abundances in summer (44.2% and 54.1% of the sequences in mesohaline and polyhaline zones, respectively), whereas sequences of the Exo-T-even group showed the highest abundances in the polyhaline zones in winter (44.0% of the total sequences). The sequences of the Freshwater Group (average, 1.4% of total sequences) and Paddy Group (average, 1.3% of total sequences) constituted a minority fraction of the viral communities in all three estuaries. The Freshwater Group and Paddy Group both showed higher abundances in the mesohaline zone than in the polyhaline zone (*P* < 0.05) as well as higher abundances in winter than in summer (*P* < 0.05). The Miscellaneous Group accounted for 8.0% to 46.6% of the total sequences in different samples. Furthermore, we found a novel clade of viral *g23* sequences from PRE, YRE, and YZRE that uniquely occurred in winter. We designated these novel sequences as Estuary-winter sequences (Fig. 4). Estuary-winter sequences showed the highest abundances in the mesohaline zones in the subtropical estuary YZRE (2.5% of total sequences), and this was followed by the polyhaline zones (2.16% of total sequences) in YZRE, with the lowest abundances being in the mesohaline zones in the subtropical estuary PRE (0.9% of total sequences) (Fig. 5).

### Shared and unique OTUs between estuaries.

**FIG 6:**
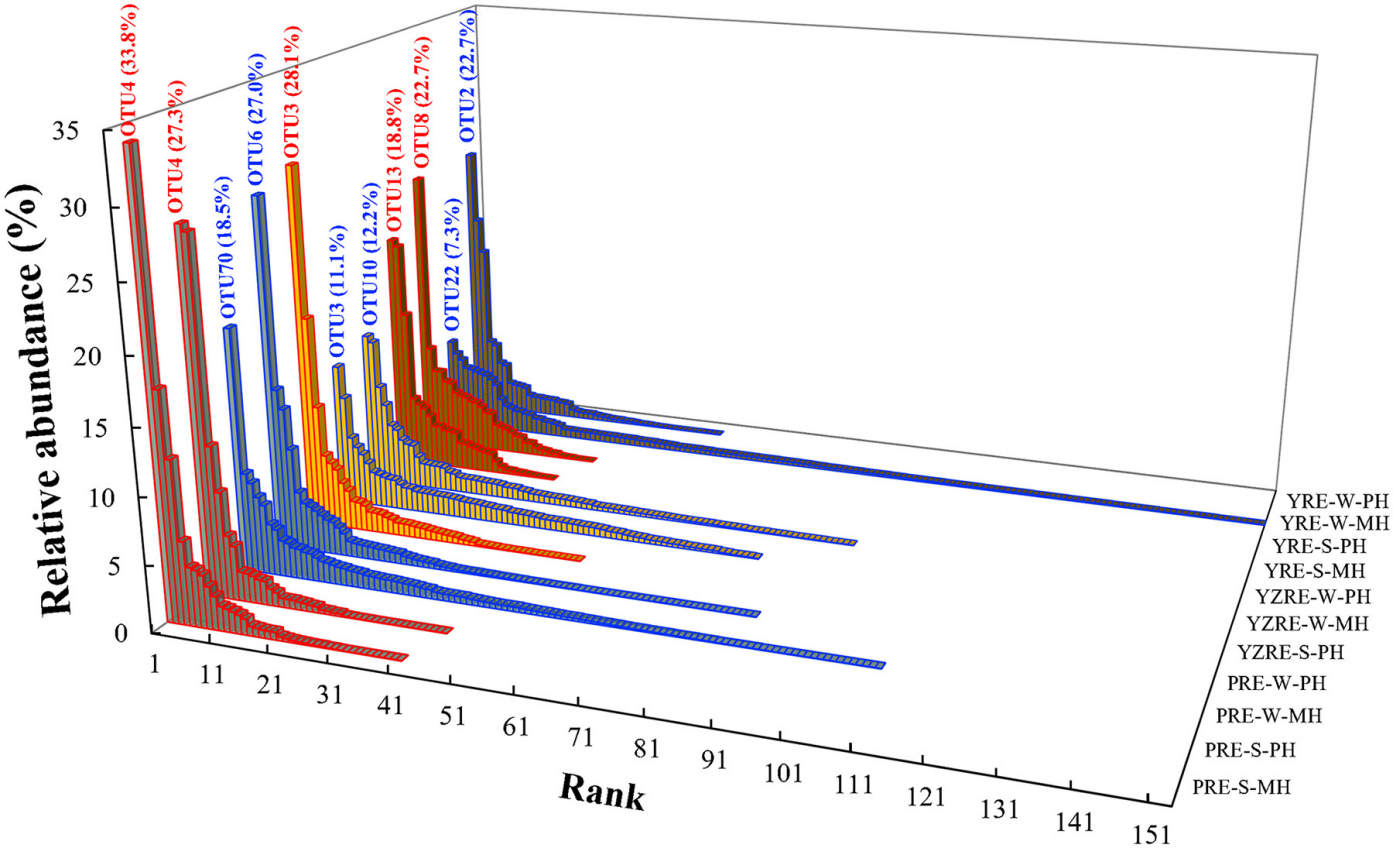
Rank abundance curves of *g23* OTUs in the estuarine samples (blue for winter and red for summer). The most abundant OTU and its relative abundance are shown next to the corresponding bar for each sample.

**FIG 7:**
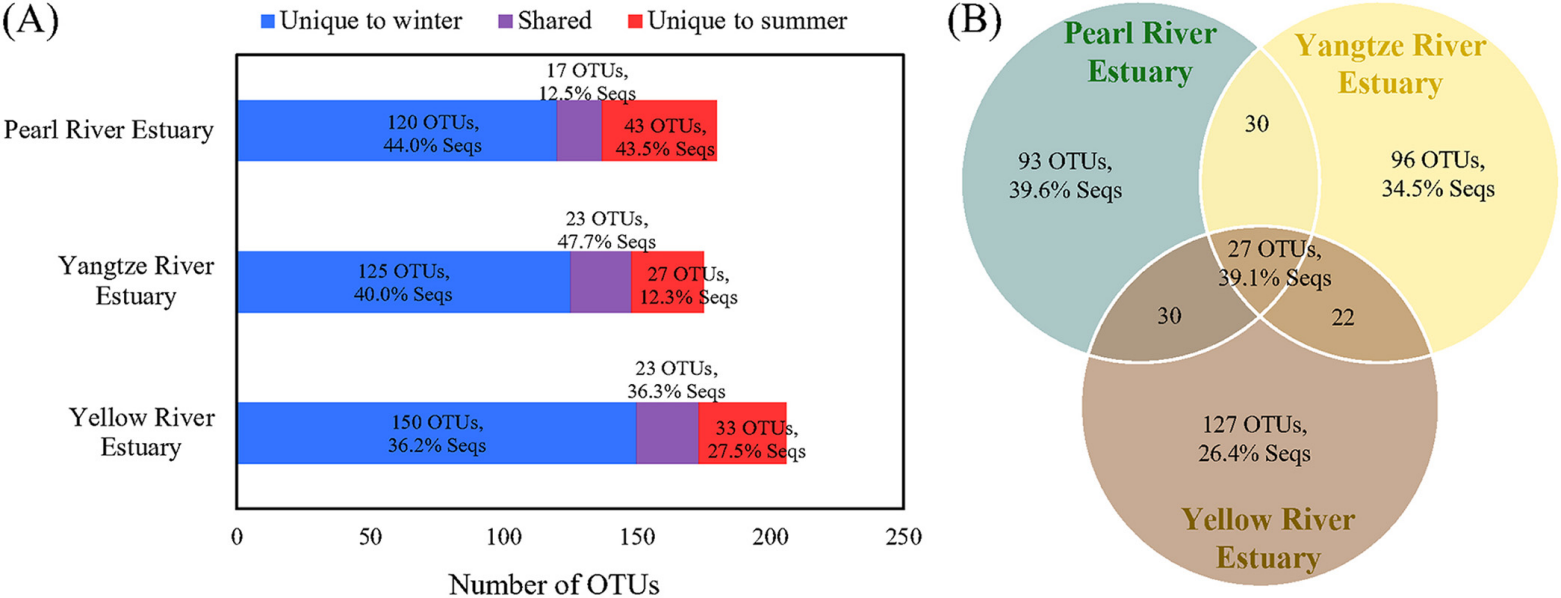
Unique and shared OTUs between different seasons and estuaries. (A) The amount of shared and unique OTUs between winter and summer in three estuaries. (B) Venn diagram showing the number of shared and unique OTUs among three estuaries. The percentages in the diagram indicate the ratios of the sequences that are associated with the OTUs.

The rank-abundance curves of OTUs for all estuarine samples were characterized by a few abundant OTUs and numerous OTUs with a relative abundance of <1% (Fig. 6). The rank-abundance curve in the winter samples showed heavier tails than those observed in the summer samples. The relative abundance of the most abundant OTUs in different samples ranged between 7.3% and 33.8% (Fig. 6). All nine of the most abundant OTUs showed the highest identities with uncultured T4-like viruses from different habitats, including the Jiulong River Estuary (18), the Pearl River Estuary (16), and the hydrothermal vents (36). The most abundant OTUs differed between estuaries but were sometimes shared by different locations and seasons within estuaries (e.g., PRE-S-MH and PRE-S-PH). To further compare the T4-like viral populations in different estuaries and seasons, the shared and unique OTUs were analyzed. Of the 425 OTUs recovered, only two OTUs (OTU16 and OTU28) that belonged to MG IV were present in all samples. More than half of the OTUs (257, 60.5%) were only present in one sample, which indicated distinct viral community compositions among samples. However, these unique OTUs had relatively low abundance, collectively accounting for only 17.0% of the total sequences in the Chinese estuaries. The number of shared OTUs between summer and winter in the three estuaries varied from 17 to 23, accounting for 12.5% to 47.7% of the sequences per estuary (Fig. 7A). Consistent with the higher diversity indices being observed in winter, the winter samples had higher numbers of unique OTUs, representing 36.2% to 44.0% of the sequences per estuary. A comparison among estuaries was performed by considering the estuary-shared OTUs to be those present in at least one sample from each estuary for all three estuaries and the estuary-unique OTUs to be those that are uniquely present in one estuary. A total of 27 OTUs (39.1% of the total sequences) were shared by the three estuaries. The OTUs that are unique to a specific estuary varied from 93 to 127, which accounted for 26.4% to 39.6% of the sequences per estuary (Fig. 7B). Most of the estuary-shared OTUs (22 out of 27) belonged to the MG, and the others were affiliated with the Miscellaneous Group.

The number of specialist OTUs ranged from 4 to 8, representing 7.6%, 29.6%, and 12.3% of the sequences in PRE, YZRE, and YRE, respectively (Fig. S4A). The specialist OTUs in PRE belonged to MG III, MG IV, and the Exo-T-even group, whereas the YRE specialists included OTUs from MG III, MG IV, the Exo-T-even group, and the Miscellaneous Group. Consistent with the result that most abundant T4-like viruses in YZRE belong to MG IV, the specialists in YZRE mostly belonged to MG IV, except for one (OTU14) from MG III. OTU3, the specialist in YZRE (Fig. S4A), was the most abundant OTU in the YZRE-S-PH and YZRE-W-MH samples and the second most abundant OTU in the YZRE-W-PH sample. Furthermore, we found that the abundance of OTU3 was positively correlated with the abundance of *Prochlorococcus* (*r *= 0.62, *P* < 0.05), which indicated that OTU3 (if it is not a cyanophage) might be closely related to the heterotrophs that coexist with *Prochlorococcus*. To identify the specific environmental factors that shaped the specialists, RDA was performed. The results showed that RDA1 explained 31.6% of the total variance, whereas RDA2 explained 22.8%. These findings reveal that the viral abundance, viral production, and temperature contributed the most to explaining the assemblies of the specialists (Fig. S4B).

## DISCUSSION

Estuarine environments are one of the most productive ecosystems on Earth, and they play a major role in global biogeochemical cycles, for which microbes are critical drivers. Microorganisms coexist with viruses that control their metabolism, activity, gene flow, and population size by infection and lysis (6, 7, 37). As viruses are the major mortality agents of microbes, delineating the viral community composition and its dynamics is essential for a complete understanding of virus-microbe interactions and the ecology of estuarine ecosystems. T4-like bacteriophages are one of the most ecologically important viral groups, and they have attracted wide attention over the past decade (9, 11, 15, 17). In this study, a spatiotemporal investigation of the T4-like viral communities was conducted, and it provided the first demonstration of the diversification and seasonality of viral assemblages in Chinese estuarine ecosystems that spanned a large range of environmental parameters.

### Diverse and novel estuarine T4-like viral communities.

A phylogenetic analysis revealed that Chinese estuarine ecosystems harbor diverse phage communities that include multiple T4-like phage subgroups. More than 90% of the sequences showed the closest identities with uncultured T4-like viruses, and most OTUs were distributed in different environmental-sequence-only clusters. These results demonstrated that a large fraction of estuarine T4-like viruses were uncharacterized as-of-yet. Obtaining the genome sequences of major viral lineages, including T4-like viruses, via isolation and other techniques (such as single-cell sequencing) has significantly improved our interpretation of viromes and PCR amplicon data (13, 38, 39). Therefore, our results highlight the need for more isolation and identification efforts for a detailed explanation of *g23* amplicon data and for the in-depth characterization of ecologically important estuarine viral groups (e.g., the most abundant OTUs in different estuaries and the Cluster I that is specific to the Chinese estuaries).

Estuarine microbial populations are generally considered to be derived from the open sea and from river discharge. Despite the significant variations in composition across sites, MG was always the most abundant T4-like viral group in the different Chinese estuaries. The OTUs that were most frequently recorded in the Chinese estuarine ecosystems generally belonged to MG III, MG IV, and the cyanophages (Exo-T-evens). Consistently, the two OTUs that were present in all samples both belonged to MG IV. MG IV has been revealed to be widely distributed in various aquatic environments, such as the Arctic glaciers, the northeastern Gulf of Mexico, and the Pacific Ocean (9). We showed that the phages in MG IV were also ubiquitous in estuarine ecosystems. In particular, MG IV was the most abundant subgroup in the subtropical estuary YZRE, regardless of the season and location. This indicated that MG IV might prefer midlatitude areas. It is interesting that MG III showed the highest relative abundances in summer in the subtropical estuary PRE but was the most prevalent in winter in YRE (in both the mesohaline and polyhaline zones in both estuaries). In previous studies, MG III was considered to be abundant in lower-salinity continental or eutrophic environments (9, 17). However, the geographic distribution of MG III in different estuarine environments indicates that their distribution may not be limited by salinity or temperature. Furthermore, our investigation revealed a unique Cluster I T4-like viral community in the Chinese estuaries. Together with the results of previous studies in the Jiulong River Estuary (18), Pearl River Estuary (40), Delaware Bay, and Chesapeake Bay (33), these results indicated that viruses might develop specific and stable populations in spatially and temporally dynamic estuarine environments. Our study also identified several previously unknown taxa that were rare in the estuarine environment. For example, Estuary-winter sequences were only present in all three estuaries in winter. However, without host and virus isolation work (see the discussion above), it is difficult to identify the microbial hosts of the Cluster I and Estuary-winter groups of viruses based on the *g23* sequences. Further microbe-virus analyses or network analyses that are based on high-resolution spatial and temporal sampling will help us predict their hosts.

T4-like virus community observations in all estuarine samples showed a small portion of abundant viral populations and a majority of rare populations. This phenomenon supports the seed-bank model, which states that only a few abundant viruses are active, whereas the majority (the bank) exist in an inactive status, which thereby results in a diverse and uneven community structure in the environment (41, 42). Under the seed-bank theory, the members forming a reservoir at low abundances can become active when the conditions are right (e.g., their hosts reach appropriate abundances). Similar seed-bank patterns were observed for marine virioplankton on a global scale (43) and for specific viral groups in both fresh and seawater ecosystems (44). However, different from the high local diversity but limited global diversity as well as from the mostly shared viral community for marine virioplankton, the estuarine T4-like viruses showed high local viral diversity and few shared viral communities (Fig. 7). Our study showed that the most dominant OTUs usually differed among samples and that there was a distinct segregation of T4-like viral community compositions across estuaries. The viral assemblage dynamics may explain previously observed variations in viral production in these environments (45), and they are likely to have been derived from changes in the host abundance, diversity, productivity, and physicochemical conditions.

### Spatial variations of within-estuary T4-like viral communities.

Our study suggested that the environmental gradients between or within estuaries create variable ecological niches for virioplankton. Spatially varied and seasonally fluctuated water temperature was the most important variable affecting the biogeography of the T4-like viruses in the estuary regions (e.g., the seasonality of viral communities in each estuary and the spatial separation of the viral communities among estuaries). Even though no significant differences were found in the viral community diversity and composition between regions with different salinity levels in an individual estuary, high relative abundances of viruses with riverine and terrestrial origins (the Freshwater and Paddy Groups, respectively) were shown in the mesohaline zones, whereas their proportions were generally relatively small or almost nonexistent in the high salinity areas (i.e., the polyhaline zones) (Fig. 5). Generally, microbes are sensitive to changes in ionic strength; the variation in salinity influences the physiological states of microbial cells (46, 47). Previous studies reported that marine bacteria showed different levels of adaptability to the estuarine environment (48). High salinity seemed to have a strong inhibitory effect on riverine bacteria (49–51), which may inhibit viral production in cells that are derived from upstream estuarine regions. Significant changes in the microbial community structure along environmental gradients were reported for these three estuaries (52–54). The close nature of the virus-microbe relationship makes it reasonable that shifts in the microbial community composition could lead to corresponding changes in the coexisting viral communities. Additionally, the viruses themselves appear to have some ionic demands to retain their structural stability. For example, optimal Na^+^ and Mg^2+^ concentrations are required for phages to maintain their high infection abilities (55, 56). Thus, the effect of the variation of the ionic strength on the decay of nonindigenous viruses (in terms of losing infectivity and particle integrity) may also be important in estuarine environments. This possibly results in higher decay rates for riverine viruses than for marine viruses in estuarine environments, which leads to low abundances of riverine-derived viruses in estuaries. With the increased salinity, riverine viruses are more sensitive to being degraded in polyhaline environments than they are in mesohaline environments. In addition, changes in salinity and ionic strength can also impact extracellular enzyme activity (such as proteases and nucleases), which contributes to viral decay via hydrolyzing viral protein capsids and internal nucleic acids (57–60). Therefore, the influence of environmental gradients on virus-host coupling, viral production, and decay may represent potential mechanisms that promote variation in the viral diversity and community structures within estuaries.

### Higher T4-like viral diversity in winter.

Seasonal variations in viral community composition were observed in the Chinese estuaries. This finding was consistent with the results of previous studies in other aquatic environments. For example, seasonally recurring patterns in viral communities were reported in the Chesapeake Bay (61), Red Sea (62), Sargasso Sea (63), and freshwater environments (64, 65). The covariation of the abundance and the genetic diversity of different host-virus pairs (e.g., *Synechococcus* and cyanophages as well as Roseobacteria and roseophages) in seasonal transitions have been previously observed in different environments (e.g., the Chesapeake Estuary) (66–68). Distinct seasonal patterns of bacterial abundance and community structure were reported in the three largest estuaries of China (45, 52–54). The shift in the bacterial communities and different prokaryotic activities between winter and summer may be the driving force of the seasonal transition of the T4-like viral communities in the three estuaries. Our observations indicated that the high levels of seasonality could thus be a common pattern for the viral community composition in natural ecosystems, including complex and dynamic estuarine ecosystems, which are mainly driven by biological parameters (e.g., microbial population size, community composition, and activity) as well as weather-related seasonal fluctuations of abiotic variables, such as temperature.

Moreover, we observed higher viral diversity in winter than in summer. This is interesting because there were lower viral abundance and production value observed in winter in these estuaries (45). This finding indicated that there were viral populations with different life history traits (e.g., host range and burst size), especially active viral populations, in different seasons. Additionally, this could be the result of viral adaptations to the seasonally changing microbial communities. Owing to the host-specific natures of viruses, the higher diversity of bacterial communities in winter may be the main reason for the higher α-diversities of the T4-like phages that were observed in winter in the three estuaries. Indeed, the α-diversities of the bacterial communities from YZRE, YRE, and PRE were also significantly higher in winter than in summer, despite the lower bacterial abundances in winter (52–54). Enhanced vertical mixing and a higher level of nutrients in winter, especially phosphorus, can trigger the activation of some dormant bacterial taxa (69, 70), which makes the bacterial community more complex and further promotes the proliferation of their corresponding phages (70, 71). For example, higher *Synechococcus* diversity has been observed in winter, compared with summer, in PRE (72), which is consistent with our observation of the higher relative abundance of cyanophages in winter in PRE (Fig. 4 and 5).

### Conclusion.

Only a few studies have previously investigated viral assemblages in estuarine ecosystems, and little is known about the differences in viral community composition across estuaries and seasons. This study is the first to analyze viral communities in different estuaries, ranging from subtropical to cold temperate climates and across seasons so as to obtain an overview of the T4-like viral communities in the three largest estuaries in China. A global analysis of all samples indicated the adequate recovery of T4-like viral diversity in the Chinese estuarine ecosystems. The results demonstrated that the T4-like bacteriophages are diverse and differ substantially across estuaries. Analyzing the diversity trends of the viral communities revealed seasonal shifts that likely coincide with the changes in the bacterial community and water temperature. Our results reinforced the idea that there exist heterogeneity and seasonality of viral assemblies in estuarine ecosystems, and they emphasized the importance of replicated experimental designs over space and time when studying estuarine viral communities in the future.

## MATERIALS AND METHODS

### Study area and sampling.

The Yangtze River is the longest river in Asia and globally ranks as the fourth largest river in terms of water discharge (73). The Yangtze River is considered a to be boundary that separates the hot and wet climate in the south from the cool and dry climate in the north in China (74). YZRE is located in eastern China, adjacent to the East China Sea. The Pearl River is the second largest river in China in terms of water discharge (73). PRE is a subtropical estuary that connects the Pearl River and the South China Sea. The Yellow River is the largest river in northern China. YRE is characterized by a temperate climate with two distinct seasons: a warm, wet summer and a cold, dry winter (73). Surface waters in the mesohaline (salinity ca. 10) and polyhaline (salinity ca. 25) zones of three estuaries were sampled in both winter (January of 2016) and summer (August of 2016). At each site, approximately 5 L of surface water were collected and prefiltered through a 20 μm mesh into acid-cleaned polycarbonate bottles. Water samples were filtered using tangential flow filtration with a 0.22 μm pore-size polyvinylidene difluoride cartridge (Millipore, USA) to remove prokaryotes and a 30 kDa polysulfone cartridge (Millipore, USA) to generate a 50 mL viral concentrate (75). The viral concentrates were stored at 4°C in the dark until further analysis in the lab.

The environmental and biological parameters of each site have been described in detail in a previous study (45), and these parameters varied based on the location and the season in the three Chinese estuarine ecosystems. Seasonally, the summer samples were characterized by significantly higher pH value, higher abundances of viruses and picoplankton, and higher viral production. Winter had significantly higher levels of dissolved oxygen, virus-to-prokaryote ratios (VPRs), and percentages of microbes that were lysed by viruses. Spatially, the polyhaline zone showed significantly higher pH values, viral abundances, VPRs, and viral production (45).

### DNA extraction, PCR amplification, and sequencing.

Before DNA extraction, the viral concentrates were treated with 1 μg/mL DNase I at room temperature for 1 h to remove the free DNA. Then, the DNase was inactivated at 65°C for 15 min. Viral particles were lysed with proteinase K (0.2 mg/mL final concentration), 0.5 M EDTA, and sodium dodecyl sulfate (0.5% wt/vol final concentration) at 55°C for 3 h. The DNA was extracted via the phenol-chloroform/isoamyl alcohol method (31). The fragments of the *g23* genes, which encode the major capsid proteins of the T4-like viruses, were amplified using the forward MZIA1bis primer (5′-GATATTTGNGGNGTTCAGCCIATGA-3′) and the reverse MZIA6 primer (5′-CGCGGTTGATTTCCAGCATGATTTC-3′), as described in the work of Filée et al. (9). The reaction conditions of amplification consisted of an initial denaturation step of 98°C for 2.5 min and 30 cycles of 45 s of denaturation at 98°C, 35 s of annealing at 53°C, 35 s of extension at 72°C, and a final elongation of 10 min at 72°C (16, 17, 20). The amplified PCR products were separated via 1.5% low-melting gel electrophoresis and were extracted with a Wizard SV Gel and PCR cleanup system. A Qubit fluorometer (Invitrogen, USA) was used to quantify the PCR products. On the basis of quality checks of the DNA extraction and the PCR amplification, the summer sample collected from the mesohaline zone of YZRE could not be successfully amplified with high-quality *g23* PCR products. Thus, it was excluded from the analysis. Equal amounts of the PCR products for each sample were pooled and sequenced using a 454 GS FLX platform (Roche Applied Science, USA).

### Sequence and statistical analyses.

The raw data were quality screened using Mothur (v1.48.0) (76) by removing sequences that: (i) had lengths of <300 or >800 bp, (ii) contained >0 ambiguous bases, or (iii) had a homopolymer length of >8 bp. Usearch (v11) was used to remove chimeric sequences and to perform operational taxonomic unit (OTU) clustering at 97% similarity with the UPARSE pipeline, using the default parameters (77). The reference *g23* sequences from the isolated phage genomes were retrieved from GenBank. BLASTP was used to examine each OTU against the reference *g23* sequences. Those with an identity value of lower than 30% were considered to be nonspecific amplifications and were removed from further analysis.

The α-diversity index for each sample was obtained using Usearch. Rarefaction curves based on the identified OTUs were estimated in R (v4.1.2) with the vegan package. Paired *t* tests were used to compare the α-diversity differences between the estuaries and the seasons. Community analyses were performed using the vegan package in R. The dissimilarity and distance matrices were calculated via the Bray-Curtis method (78). Bray-Curtis dissimilarities are useful for quantifying the differences in the community composition between sites (78). For example, if the two sites have the same composition (i.e., they share all species), the Bray-Curtis dissimilarity equals 0. In contrast, if two sites have high dissimilarity in that they share almost no species, the dissimilarity value will be closer to 1. Nonmetric multidimensional scaling (NMDS) ordination was performed to visualize the differences in the viral communities among the samples. A nonparametric multivariate analysis of variance (PERMANOVA) was used to determine the statistically significant differences between estuaries or seasons, based on the Bray-Curtis dissimilarity with 999 permutations. Mantel tests were conducted to analyze the contributions of environmental variables to the viral community. To identify the OTUs that were specific to a given estuary, we used the indicator species analysis proposed by Dufrêne and Legendre (79). This analysis determines indicator species by combining the relative abundance (specificity) with the relative frequency of occurrence (occupancy) in the habitats. Here, specificity was defined as the mean relative abundance of an OTU in a given estuary, relative to the sum of the relative abundances of the OTU in all three estuaries. Occupancy was defined as the number of sites where the OTU occurred, relative to the number of sites in a given estuary. OTUs with specificity and occupancy values of ≥0.7 were selected as specialists (i.e., they are specific to one estuary and are common in most sites in that estuary), following the methods of Gweon et al. (80). A redundancy analysis (RDA) was performed to evaluate the relationships between the special OTUs and the environmental variables. Prior to the RDA, a detrended correspondence analysis was conducted to calculate the gradient lengths. The protein sequences were aligned using MUSCLE (v3.8.1551) (81), and maximum likelihood phylogenetic trees were built using IQ-Tree (v1.6.6), using the auto-detected models and a maximum iteration of 1,000 (82, 83). The Interactive Tree Of Life (iTOL) was used to visualize and edit the tree (84).

### Data availability.

The raw data of the sequences have been deposited in the National Center for Biotechnology Information Sequence Read Archive under the BioProject accession number PRJNA911213 with the BioSample accession numbers SAMN32183246 to SAMN32183256.
