# Supplementary material for: Spatiotemporal Shift of T4-Like Phage Community Structure in the Three Largest Estuaries of China
Source: Microbiol Spectr. 2023 Mar 6;11(2):e05203-22. doi: 10.1128/spectrum.05203-22 (PMC10101079; doi:10.1128/spectrum.05203-22)
Supplement: Supplemental file 1 — Fig. S1 to S4. Download spectrum.05203-22-s0001.pdf, PDF file, 0.6 MB [file spectrum.05203-22-s0001.pdf]

Supplementary Information for

Spatiotemporal shift of T4-like phage community structure in three largest estuaries of China

Lanlan Cai, Bu Xu, Huifang Li, Yongle Xu, Wei Wei, Rui Zhang

Corresponding author: Rui Zhang (ruizhang@xmu.edu.cn)

This file includes: Figures S1 to S4

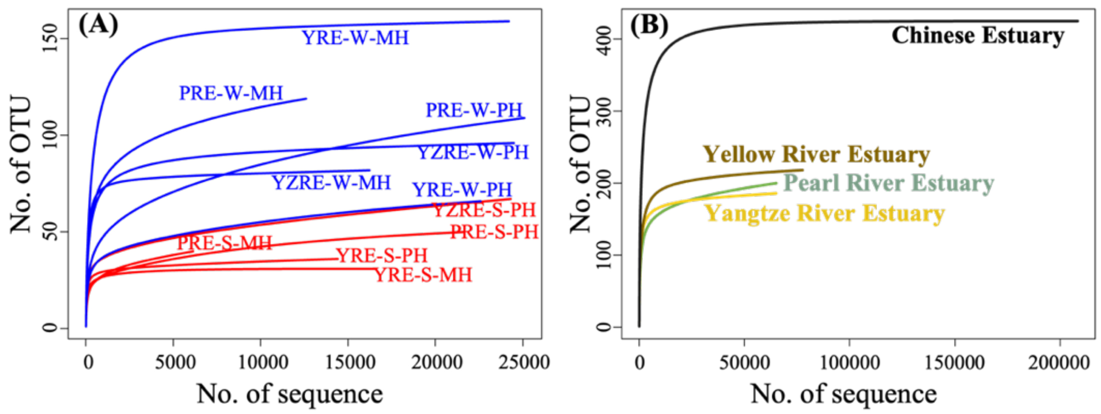

Figure S1. Rarefaction analysis of *g23* OTU diversity for each sample (A) and the Chinese estuaries as a whole (B). Sample name was presented in the format of estuary-sampling season-salinity level, with S standing for summer and W for winter. PRE, Pearl River Estuary; YZRE, Yangtze River Estuary; YRE, Yellow River Estuary; MH, mesohaline zone; PH, polyhaline zone. Each sample is colored based on the sampling season (blue for winter and red for summer).

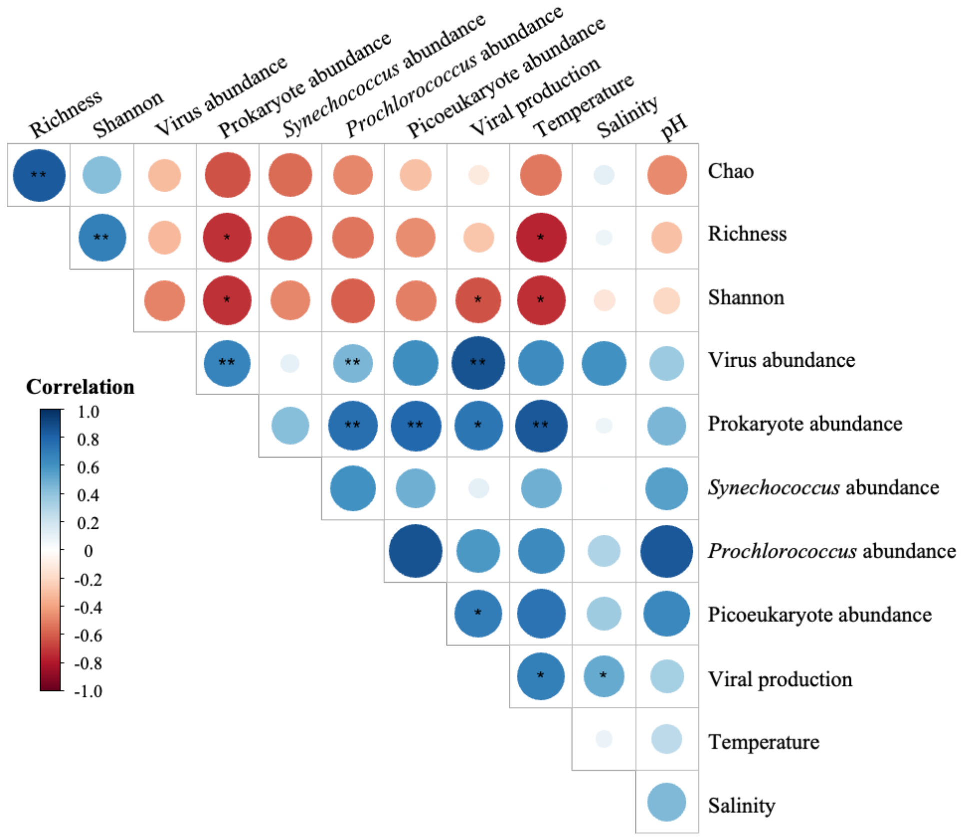

Figure S2. Spearman correlation coefficients among microbial and environmental factors in three Chinese estuaries. Pairwise comparisons with a color gradient denoting Spearman's correlation coefficient and p values were shown in the circles (*, p<0.05; **, $p < 0.01$; ***, $p < 0.001$).

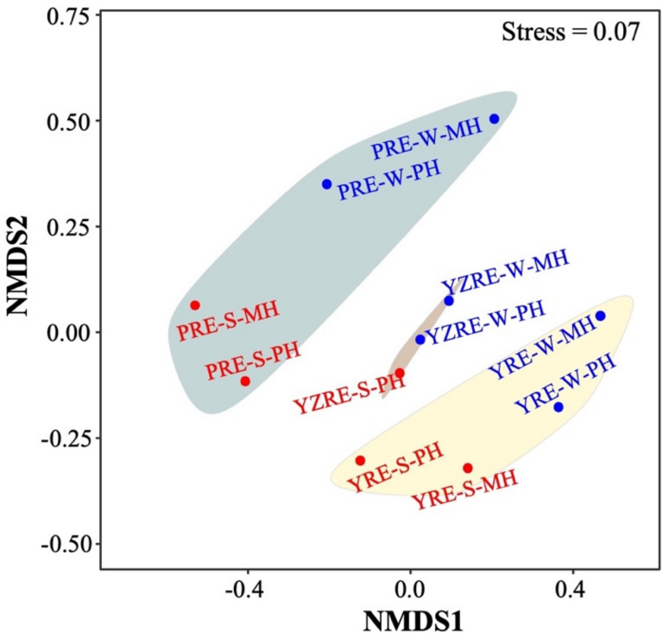

Figure S3. Nonmetric multidimensional scaling (NMDS) plot showing the variation in T4-like viral community composition between estuaries. Pairwise community distances were determined using Bray-Curtis dissimilarities at the OTU level. Stress level is indicated. Samples (points) are colored based on sampling season (blue for winter and red for summer). Significant differences between estuaries were verified by PERMANOVA ($p \leq 0.05$).

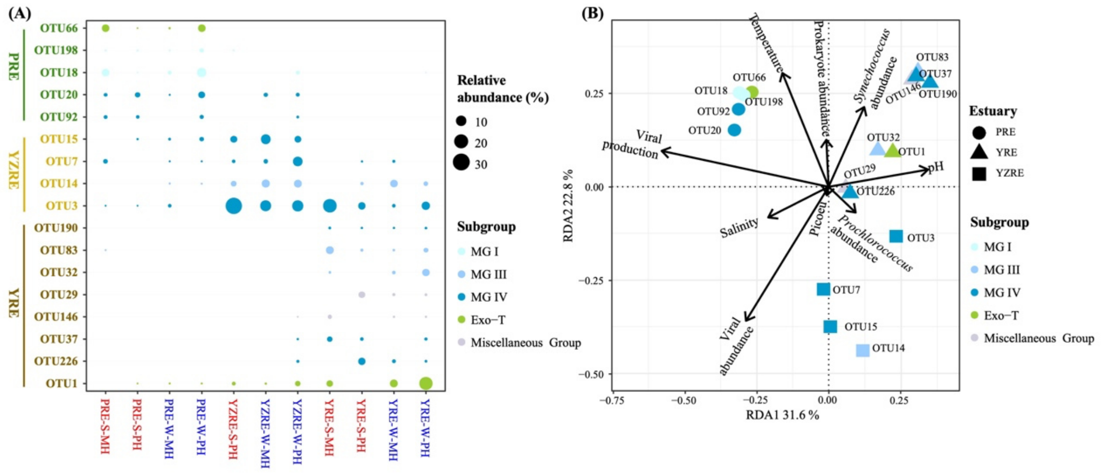

Figure S4. (A) Relative abundance bubble plot of the specialist OTUs for three estuaries. The colors of the bubbles indicate the taxonomy of OTUs, and the sizes correspond to the relative abundance of OTUs in each sample. (B) Redundancy analysis ordination diagram of specialist OTUs in different estuaries and environmental variables. Dots indicate OTUs. The arrow lengths and directions correspond to the environmental variances. The angles between OTUs and environmental factors denote their degree of correlation.
